# The core self-evaluations, psychological capital, and academic engagement: a cross-national mediation model

**DOI:** 10.3389/fpsyg.2023.1189665

**Published:** 2023-07-19

**Authors:** Ramona Paloș, Elena Mirela Samfira, Delia Vîrgă, Daliborka Purić

**Affiliations:** ^1^Department of Psychology, West University of Timișoara, Timișoara, Romania; ^2^Teacher Training Department, University of Life Sciences “King Mihai I” from Timișoara, Timișoara, Romania; ^3^Faculty of Education in Užice, University of Kragujevac, Kragujevac, Serbia

**Keywords:** core self-evaluations, psychological capital, academic engagement, personal resources, higher education

## Abstract

**Introduction:**

An academic environment with continuously more demanding tasks requires students to capitalize on their strengths to meet the challenges and engage in learning experiences. Engaged students are deeply involved in their work, are strongly connected with their studies, and are more successful in academic tasks. The present study aimed to test a model in that core self-evaluations (CSE) predicts academic engagement (AE) directly and indirectly by increasing personal resources (i.e., psychological capital; PsyCap) in the case of two different samples, Romanian and Serbian.

**Methods:**

Data were collected through three online questionnaires from 672 undergraduate students (Romania – 458; Serbia – 214).

**Results:**

The findings confirmed that CSE was positively related to PsyCap, which was positively associated with AE, and PsyCap mediates the relationship between the two variables in both samples. A positive evaluation of one’s characteristics (high CSE) mainly affects the cognitive and emotional mechanism of appraising the academic-related tasks one encounters (high PsyCap), ultimately shaping their motivation and engagement.

**Discussion:**

These results pointed out the importance of the CSE and PsyCap that support each other and increase students’ AE, explaining the mediating mechanism of PsyCap. Also, they provide insight into the students’ engagement from two different cultural and educational contexts, being helpful to universities in their effort to increase students’ engagement.

## 1. Introduction

An academic environment with increasingly demanding assignments requires students to capitalize on their strengths to meet challenges and engage in learning experiences (Bowden et al., 2021). Academic engagement (AE) is seen as an „intermediate outcome” that facilitates learning (Choi and Rhee, 2014) and is linked to students’ intrinsic motivation, perseverance in academic activities, higher educational aspirations, and success (Lei et al., 2018; Ketonen et al., 2019; Paloş et al., 2019). Engaged students are deeply involved in their work, feel energized, are strongly connected with their studies (Martínez et al., 2019), and are more successful in academic tasks (Schaufeli et al., 2002). Earlier studies found that AE is essentially shaped by personality (Siu et al., 2014; You, 2016; Tisu et al., 2020). For instance, recent research highlighted that AE is strongly predicted by students’ core self-evaluations (CSE; Yan et al., 2018), a trait-like personality characteristic that reflects people’s assessment of themselves and their self-worth (Judge et al., 2003). Prior studies illustrated that high CSE people tend to assess situations positively, are confident in their capacity to succeed, are highly motivated to value opportunities, and are more effective in self-regulation (Bipp et al., 2019), CSE being a strong predictor of AE (Yan et al., 2018). Also, psychological capital (PsyCap), a state-like personality characteristic reflecting “an individual’s positive psychological state of development” (Luthans et al., 2007, p. 542), is a significant antecedent for engagement (Siu et al., 2014; Fang and Ding, 2020). Considered at the same time as a malleable personal resource that increases learning engagement (You, 2016; Alessandri et al., 2018), PsyCap stimulates various other resources that can successfully support the boost of new personal resources (Robayo-Tamayo et al., 2020). According to the Conservation Resources theory (COR; Hobfoll and Ford, 2007), resources are seen as internal and external strengths that people can use to cope with and adapt to challenging situations (Hobfoll and Ford, 2007) and are essential for motivation and goals attainment (Hobfoll et al., 2018). People use resources (e.g., personal, social) not only to deal with difficult situations but also to increase the pool of resources for future challenges. They do not exist individually, they are interrelated and form a “caravan of resources” that support and enhance each other (Hobfoll, 2011). Hence, available resources influence people’s capacity to obtain more resources (Hobfoll, 2011; Robayo-Tamayo et al., 2020). The more personal resources they have, the more they can gain, which sustains them to follow their goals and engage in different activities (Ouweneel et al., 2011; Martínez et al., 2019; Ma et al., 2022).

**Figure 1:**
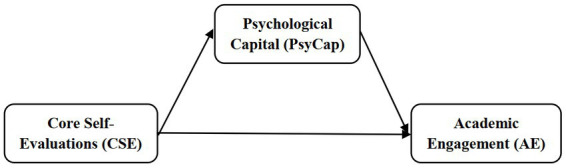
Hypothetical model of the research.

Engagement and personality (i.e., CSE and PsyCap) were mainly explored in the organizational environment (e.g., Bipp et al., 2019; Tisu et al., 2020), but only a few of the studies were carried out in the academic areas (e.g., You, 2016; Leupold et al., 2020). To fill this gap, the present research was performed in the educational context and *aimed to test a model in that CSE predicts AE directly and indirectly by increasing personal resources* (i.e., *PsyCap*) *in the case of two different samples, Romanian and Serbian* (Figure 1). Our study can contribute new insights into theory. First, based on COR theory, it expands the knowledge regarding the relationships between two personality characteristics and AE for students in Higher Education. On the one side, CSE is a trait-like personality characteristic resistant to change; on the other, PsyCap is state-like and malleable (Howard, 2017). Investigating their relationship with AE helps us better understand individual differences (Tisu et al., 2020) and how PsyCap can be trained to increase student engagement in Higher Education. Second, the model was tested in two different samples among students in Higher Education, Romanian and Serbian, to explore the cross-national validity of our results.

### 1.1. CSE and PsyCap

CSE was described as a personality trait that assesses an individual’s competence, effectiveness, capabilities, and worthiness and includes four components: self-esteem [a general “appraisal of one’s self-worth” (Rosenberg, 1965)], generalized self-efficacy [the people’s belief that they can perform complex tasks or cope with an aversive situation in diverse areas (Schwarzer and Jerusalem, 1995)], emotional stability [the predisposition “to feel calm and secure” (Eysenck, 1990)], and locus of control [the conviction that wanted consequences are the result of the behavior rather than luck, faith, or others (Judge et al., 2003)]. People high in CSE are more confident in their abilities, can deal with different demands, and have more available resources to solve problems (Judge and Hurst, 2007). Also, they evaluate various situations more positively and are confident in their capacity to control things and achieve goals (Ma et al., 2022). CSE is linked to PsyCap, a state-like psychological attribute involved in assessing the self in relation to the environment (Howard, 2017), and also a valuable personal resource (Siu et al., 2014). PsyCap is a multidimensional construct composed of self-efficacy (the confidence that one can fulfill a demanding task), optimism (the belief that one can succeed, now and in the future), hope (persistence in achieving aims and redirecting paths if obstacles appear), and resilience (one’s ability, when encountering difficult situations, to bounce back from challenges or failures) (Luthans et al., 2007). The four elements act synergistically to increase people’s implication in different domains of their life (Gibson and Hicks, 2018; Sava et al., 2020). Previous research showed CSE as an antecedent for PsyCap—a motivational potential that provides toughness to attain success (Luthans et al., 2007; Luthans and Youssef-Morgan, 2017), individual differences being the strongest predictor of PsyCap. Moreover, individuals with a high level of CSE proved to be more effective in generating resources to help them be more motivated and involved in tasks (Bipp et al., 2019). Based on the above arguments, the first hypothesis was developed:

*H1*: CSE is positively associated with PsyCap.

### 1.2. PsyCap and AE

Seen as a “state of fulfillment characterized by vigor, dedication, and absorption” (Schaufeli et al., 2002, p. 74), AE was described as mental energy which could generate students’ enthusiasm and motivation for their educational activities (Stoeber et al., 2011). Engaged students are curious and interested, are open to challenges and enjoy complex tasks, are more persistent and tenacious (Shih, 2008), self-efficacious with a good sense of belonging (Kahu et al., 2020) and feel deeply involved in flow conditions (Shernoff et al., 2003). According to previous studies, PsyCap is a motivational potential that supports engagement (Firouznia et al., 2021). Students with a high PsyCap are more intrinsically motivated, actively engaged in their school-related tasks, optimistic, and enthusiastic in following their goals (Datu et al., 2018; Vîrgă et al., 2020; Firouznia et al., 2021). Thus, PsyCap is a psychological resource that fosters engagement as a core construct and through each dimension taken separately (Ouweneel et al., 2011; Alessandri et al., 2018). Therefore, the second hypothesis was formulated:

*H2*: PsyCap is positively associated with AE.

### 1.3. CSE and AE: PsyCap as a mediator

People with a positive self-regard (high CSE) are more likely to develop a positive feeling about their work, perceive tasks as more attractive (Judge et al., 2003), become more desired to involve in different actions, and engage more easily (Tims and Akkermans, 2017). How people evaluate themselves (i.e., CSE) impacts how they assess the world and use their resources (i.e., PsyCap) (Howard, 2017). Thus, a high level of CSE can help students to enhance their PsyCap. Those with high PsyCap will invest more effort in achieving their goals, raising their engagement (Alessandri et al., 2018). So, students who are confident in their competencies (i.e., self-efficacy), motivated to achieve their goals (i.e., hope), determined to deal with difficult situations (i.e., optimism), and capable of adequately adapting (i.e., resilience) are more academically engaged (Siu et al., 2014; Nolzen, 2018; Carmona-Halty et al., 2021; Firouznia et al., 2021). Moreover, evidence showed mutual relationships between AE and PsyCap that can be explained through the COR mechanism (Ouweneel et al., 2011; Siu et al., 2014; Martínez et al., 2019). Hence, people’s capacity to gain more resources is influenced by their existing resources (Ma et al., 2022). When students’ resources are high (i.e., PsyCap), their repertoire of strategies to achieve the goals and overcome the encountered obstacles is richer and more diverse; they are more confident in their strengths and engage more in the study activity. Consequently, AE leads to better performance, perceived as positive feedback of competencies and invested effort. This, in turn, enhances their PsyCap by increasing confidence in their abilities, the hope that the investment of energy will help to achieve the proposed goals and that they will have the resources to overcome obstacles (Siu et al., 2014). Therefore, we anticipate that PsyCap can better explain the relationship between CSE and AE, and we assumed that:

*H3*: PsyCap mediates the relationship between CSE and AE.

## 2. Materials and methods

### 2.1. Procedure and samples

This study was carried out with participants from Romania and Serbia, which allowed us to investigate the relations between CSE, PsyCap, and AE in different educational and cultural contexts. According to the Hofstede cultural dimensions model, both are similar Balkan countries with high power distance, feminism, low individualism, high uncertainty avoidance, and short-term orientation (Hofstede et al., 2010). There are slight differences only between power distance, individualism, and indulgence (Hofstede et al., 2010; Gavreliuc, 2018).

The Romanian sample comprised 458 students (73.8% women), averaging 21.69 years (SD = 5.40). The Serbian sample consisted of 214 students (77.1% women) with an average age of 23.03 years (SD = 1.97). For both countries, the students voluntarily involved in the study were enrolled in Educational Psychology and Teacher and Preschool Teacher Education courses. They were asked to bring two other students willing to participate in the research. The participants were selected through a combination of non-probability and snowball sampling methods. Interested students got a link to a Google Forms document. The first part provided information about the study objectives, the conditions, the ethical aspects, and the voluntary character of the participation. The second part was accessible only to those who expressed their agreement and included the items of three questionnaires. The time needed to answer these items was approximately 20 min. All the procedures followed the ethical standards of the Scientific Council of University Research and Creation from West University of Timisoara (26093/05.05.2022).

### 2.2. Instruments

*CSE* was measured with the 12-item Core Self-Evaluations Scale (Judge et al., 2003). CSE is a higher-order construct made up of four interrelated traits: self-esteem (e.g., “Sometimes when I fail, I feel worthless”), generalized self-efficacy (e.g., “When I try, I generally succeed”), neuroticism (e.g., “Sometimes I feel depressed”), and locus of control (e.g., “I determine what will happen in my life”). The participants agreed with the items’ content on a 5-point Likert scale (1 = strongly disagree, 5 = strongly agree). The internal consistency was 0.84 for the Romanian and 0.78 for the Serbian samples. The questionnaire was previously used in further research in both countries (e.g., Ivanović and Ivanović, 2018; Tisu et al., 2020).

*PsyCap* was measured with the 24-item Psychological Capital Questionnaire (Luthans et al., 2007), with four sub-dimensions: *hope* (e.g., „Right now I see myself as being pretty successful at university”), *self-efficacy* (e.g., „I feel confident analyzing a long-term problem to find a solution”), *resilience* (e.g., „When things are uncertain for me at university, I usually expect the best”), and *optimism* (e.g., „I am optimistic about what will happen to me in the future as it pertains to studies”). The participants rated the statement on a 6-point Likert scale from *strongly disagree* to *strongly agree*. Because the four dimensions together strongly affect different variables than each taken separately (Siu et al., 2014; Nolzen, 2018), the composite score was used. Alpha Cronbach was 0.91 for the Romanian and 0.92 for the Serbian samples. The scale was adapted to be used for university students by Lupșa and Vîrgă (2018).

*AE* was measured with the 14-item Utrecht Work Engagement Scale for Students (UWES-S; Schaufeli et al., 2002). The instrument measures three dimensions: *dedication* (e.g., “My studies inspire me”), *vigor* (e.g., “When I study, I feel like I am bursting with energy”), and *absorption* (e.g., “When I am studying, I forget everything else around me”), evaluated on a 7-point like Likert scale from *never to always*. Because these three dimensions are closely related, the authors recommend using the scale’s total score (Schaufeli et al., 2006). Alpha Cronbach was 0.90 for the Romanian and 0.94 for the Serbian samples. The questionnaire was previously used in other research in both countries (e.g., Petrović et al., 2017; Paloş et al., 2019).

### 2.3. Statistical analysis

RStudio (2020) was used for data analysis. Normal distributions were presented for all variables in both samples. We used maximum likelihood estimation methods. Thus, we assessed the goodness-of-fit of the model using the *χ*^2^ test statistic, two relative fit indices (the Comparative Fit Index – CFI and the Tucker-Lewis index – TLI), also the Standardized Root Mean Square Residual (SRMR), and the Root Mean Square Error of Approximation (RMSEA), as absolute fit indices. As cut-off points, values higher than 0.90 (for CFI and TLI) or 0.08 or lower (for SRMR and RMSEA) mean a good model fit (Byrne, 2009). The Akaike Information Criterion (AIC) assessed the difference between the non-nested models. AIC with smaller values indicated a better model fit. Also, we tested the mediation model invariance across both Romanian and Serbian samples. Invariance between the compared groups is identified by a non-significant Δ*χ*^2^ statistic and a change of ΔCFI value smaller than 0.01 (Cheung and Rensvold, 2002). Indirect effects were evaluated with 95% confidence intervals using 5,000 bootstrap samples.

Therefore, the measurement and structural models were tested. First, with confirmatory factor analysis (CFA), we evaluate two measurement models: a model with one factor (M1) and a three-factor model (M2). Before testing the structural models, we used item parcels based on the factorial algorithm to optimize the indicator-to-sample size ratio and apply a latent variable approach for CSE (Rogers and Schmitt, 2004). Based on the Little et al. (2002) recommendation, each parcel should contain between three and five items. Second, two structural models that place PsyCap as a mediator have been tested: a total mediation model (M4, the hypothesized model) and a fully constrained model (M5).

## 3. Results

### 3.1. Measurement models

According to Table 1, CFA was used to compare the two measurement models for both samples. Thus, we tested M1—a single-factor model (with all observed variables loading on one latent variable for common method bias) and M2—a three-factor model (CSE, PsyCap, and AE). For the Serbian and Romanian samples, the single-factor model (M1) had not a good fit with the data, but M2 fit the data better in both samples. Thus, we restrained the three-factor model (M2). These results indicate that common-method bias is improbable to be a significant problem for both samples.

**Table 1:** Multiple group analyses (MGA) of the measurement models including the Romanian (*N* = 458) and Serbian Samples (*N* = 214).

Model	*χ*^2^	Df	*χ*^2^/df	CFI	TLI	RMSEA [90% CI]	SRMR	AIC	∆*χ*^2^	∆df
**Measurement models**										
**Romanian sample**										
M1-single-factor model	858.75**	35	24.53	0.66	0.56	0.22 [0.21, 0.24]	0.12	11288.16		
M2-three-factors model	124.49**	32	3.89	0.96	0.95	0.07 [0.06, 0.09]	0.04	10559.90	734.26	3
**Serbian sample**										
M1-single-factor model	372.81**	35	10.65	0.70	0.61	0.21 [0.19, 0.23]	0.12	5117.04		
M2-three-factors model	104.35**	32	3.26	0.93	0.91	0.10 [0.08, 0.12]	0.06	4854.58	268.43	3

**p < 0.001. *χ*^2^, chi-square; df, degrees of freedom; TLI, Tucker–Lewis index; CFI, Comparative Fit Index; RMSEA, root mean square error of approximation; CI, confidence interval; AIC, Akaike information criterion; For M2 models, the comparison is vs. M1 for each sample.

### 3.2. Preliminary results

The means, standard deviations, and inter-correlations for both samples are presented in Table 2. Alpha Cronbach takes values from 0.78 to 0.94, suggesting the acceptable reliability of the scales used in this research.

**Table 2:** Means, standards deviation, and correlation coefficients between variables for the Romanian and (*N* = 458) and Serbian (*N* = 214) Samples.

Variable	*M*_1_	SD_1_	*M*_2_	SD_2_	1	2	3	4	5	6	7	8	9	10
1. CSE	43.32	8.02	45.52	6.68	*(0.84/0.78)*	0.49**	0.49**	0.47**	0.64**	0.61**	0.34**	0.35**	0.27**	0.37**
2. Self-efficacy	4.53	1.42	4.46	0.96	0.49**	*(0.89/0.89)*	0.75**	0.60**	0.54**	0.87**	0.40**	0.40**	0.35**	0.44**
3. Hope	5.15	1.09	4.62	0.99	0.50**	0.62**	*(0.84/0.90)*	0.66**	0.58**	0.90**	0.37**	0.41**	0.29**	0.41**
4. Resilience	4.86	0.91	4.38	0.84	0.36**	0.48**	0.55**	*(0.79/0.71)*	0.53**	0.82**	0.29**	0.25**	0.15**	0.26**
5. Optimism	4.65	0.77	4.65	0.83	0.31**	0.40**	0.45**	0.45**	*(0.58/0.69)*	0.78**	0.41**	0.40**	0.36**	0.45**
6. PsyCap	4.80	0.83	4.53	0.76	0.54**	0.85**	0.84**	0.76**	0.67**	(*0.91/0.92*)	0.44**	0.43**	0.34**	0.46**
7. Vigor	2.96	1.26	3.15	1.39	0.36**	0.49**	0.54**	0.42**	0.34**	0.57**	*(0.82/0.90)*	0.54**	0.72**	0.87**
8. Dedication	4.05	1.10	4.24	1.46	0.32**	0.42**	0.61**	0.40**	0.32**	0.56**	0.69**	*(0.78/0.93)*	0.67**	0.86**
9. Absorption	3.17	1.34	3.32	1.64	0.19**	0.35**	0.46**	0.31**	0.24**	0.44**	0.76**	0.69**	*(0.75/0.89)*	0.90**
10. AE	47.79	15.49	50.33	18.27	0.33**	0.47**	0.60**	0.42**	0.33**	0.59**	0.92**	0.88**	0.90**	*(0.90/0.94)*

*N*_1_ = 458, *N*_2_ = 214, 1 = Romania, 2 = Serbia. Romanian correlations below the diagonal and Serbian correlations above the diagonal. CSE, core self-evaluations; PsyCap, psychological capital; AE, academic engagement. Cronbach’s α coefficients are presented on the diagonal: the first value is for Romanian sample and the second value is for Serbian sample. **p < 0.001. Values of the internal consistency alphas are displayed in italic in the diagonal.

**Figure 2:**
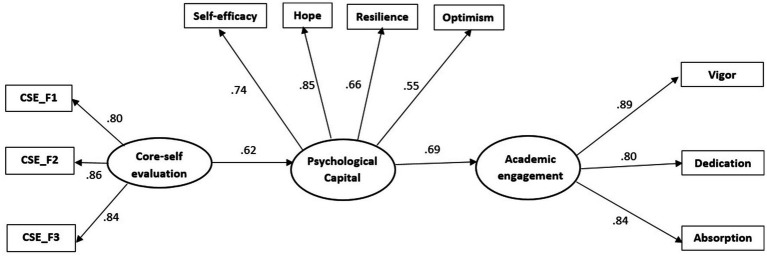
Standardized structural relations among variables from the model for the Romanian sample.

**Figure 3:**
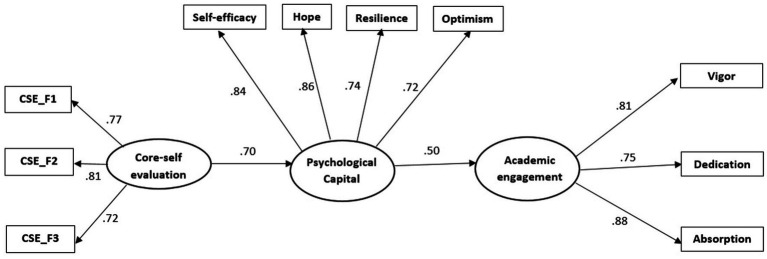
Standardized structural relations among variables from the model for the Serbian sample.

We used multiple-group SEM (Structural Equation Modeling) to evaluate whether the structural model was invariant across the Romanian and Serbian samples. The model hypothesized (M4) had good goodness-of-fit indices (*χ*^2^ (66) = 241.97, CFI = 0.95, TLI = 0.93, SRMR = 0.05, RMSEA = 0.08, 90% CI [0.07, 0.10]; see Table 3). Also, an inspection of the separate paths revealed that CSE is related to PsyCap, which is related to AE in both samples. The final model in both samples (M4) (AIC = 15455.82) is shown in Figures 2, 3. Starting from M4, one constrained model (M5) was incidental to evaluate the invariance of the model in two samples. Thus, this model had all structural parameters (relationships) constrained to be equal across samples. The fit of the constrained M5 did not significantly damage as compared to M4 (*∆χ*^2^ *=* 10.89, *n.s*.; *∆*CFI = 0.00). Thus, the relationships between the three observed variables specified in M4 are invariant across the samples (Romanian and Serbian).

**Table 3:** Multiple group analyses (MGA) of the structural models including the Romanian (*N* = 458) and Serbian samples (*N* = 214).

Model	*χ*^2^	Df	*χ*^2^/df	CFI	TLI	RMSEA [90% CI]	SRMR	AIC	∆*χ*^2^	∆df	∆CFI
**Structural model**											
M4-hypothetical model	241.97**	66	3.66	0.95	0.93	0.08 [0.07, 0.10]	0.05	15455.82			
M5-full constrains model	252.86**	68	3.71	0.95	0.93	0.09 [0.07, 0.10]	0.05	15462.71	10.89	2	0.00

**p < 0.001. *χ*^2^, chi-square; df, degrees of freedom; TLI, Tucker–Lewis index; CFI, Comparative Fit Index; RMSEA, root mean square error of approximation; CI, confidence interval; AIC, Akaike Information Criterion.

### 3.3. Testing the hypotheses

#### 3.3.1. CSE and PsyCap

*H1* stated that CSE is positively associated with PsyCap. The findings presented in Figure 2 supported *H1*. CSE was positively related to PsyCap in both samples (*β* = 0.62, *p* < 0.001 for the Romanian sample and *β* = 0.70, *p* < 0.001 for the Serbian sample). We obtained a stronger association between CSE and PsyCap when applying the constraints of the relationship to be equal for both samples (*β* = 0.84, *p* < 0.001).

#### 3.3.2. PsyCap and AE

*H2* established that PsyCap is positively associated with AE (*β* = 0.69, *p* < 0.001 for the Romanian sample and *β* = 0.70, *p* < 0.001 for the Serbian sample). Figures 2, 3 illustrate that the results supported *H2*. PsyCap was positively related to AE. We obtained comparable results but lower for each of the two samples (*β* = 0.63, *p* < 0.001) when limiting the relationship between PsyCap and AE to be equal for both samples.

#### 3.3.3. PsyCap as a mediator

Related to *H3*, PsyCap acts as a mediator in the relationship between CSE and AE. The results supported the mediating role of PsyCap in both samples based on bootstrapping techniques. For the Romanian sample, the indirect path linking CSE to AE via PsyCap was 0.59 (CI 95% [0.46; 0.73]) and 0.40 (CI 95% [0.26, 0.55]) for the Serbian sample. In both samples, the result indicates that a high CSE is related to PsyCap and with high AE. Also, in M5, we tested an indirect path between the two samples, achieving the same effect of 0.53 (95% CI [0.47; 0.64]). Figures 2, 3 illustrate that PsyCap fully mediated between CSE and AE (*H3* is supported). Thus, for the Romanian sample, the explained variance in the mediator, PsyCap (*R*^2^ = 0.48), and the outcome, AE (*R*^2^ = 0.48), was relatively like for the Serbian sample (*R*^2^ = 0.49 for PsyCap, and it is less *R*^2^ = 0.25 for AE).

## 4. Discussion

The research aimed to test if CSE *predicts AE directly and indirectly by increasing personal resources* (i.e., *PsyCap*) *in the case of two different samples, Romanian and Serbian*. Hence, the results showed positive relationships between CSE, PsyCap, and AE for both students’ samples, emphasizing the mediator role of PsyCap. In other words, students with positive CSE are more confident in their capacity to control things, deal with academic challenges, look for and be involved in exciting and complex tasks, and set ambitious goals (Gibson and Hicks, 2018). They feel in control of their academic activities’ outcomes due to their abilities and effort (Gibson and Hicks, 2018; Ma et al., 2022). Experiencing the satisfaction of good results acts as positive feedback that supports students’ self-efficacy, hope, and optimism (Ouweneel et al., 2011). Therefore, their positive evaluations (high CSE) shape how they assess academic demands, mobilize, and use resources to meet these requirements (Howard, 2017). Thus, a positive evaluation of one’s characteristics (high CSE) mainly influences the cognitive and emotional mechanism involved in the appraisal of the academic-related tasks they encounter (high PsyCap), ultimately shaping their motivation and engagement (Chang et al., 2012). So, according to the COR mechanism, CSE supports the use of existing resources (i.e., PsyCap) that can lead to a more significant investment of effort and engagement in academic tasks and act as a reservoir from which students can take or add other resources (Alessandri et al., 2018; Martínez et al., 2019; Firouznia et al., 2021).

The data sustained all the hypotheses, and our results are aligned with previous studies conducted in an organizational and educational context (e.g., Datu et al., 2018; Gibson and Hicks, 2018; Martínez et al., 2019). For instance, Bipp et al. (2019) found that CSE is essential in generating resources to support engagement. Although CSE is considered a trait-like characteristic, recent empirical findings showed room for change which has crucial practical implications. Leupold et al. (2020) state that self-esteem and self-confidence can be increased through cognitive and behavioral intervention, leading to high CSE. Also, the level of neuroticism can be decreased by strengthening the overall CSE and PsyCap (Gibson and Hicks, 2018). The role of PsyCap in increasing students’ AE is also emphasized by former research (Luthans et al., 2016; Vîrgă et al., 2020). As a state-like personal resource, PsyCap can be increased through training and coaching sessions (Luthans and Youssef-Morgan, 2017; Lupșa et al., 2020). Developing students’ PsyCap can improve their AE and, finally, their performance (Lupșa et al., 2020).

From the cultural perspective, the present research showed that the relationships between CSE, PsyCap, and AE are relevant for Romanian and Serbian university students. These results align with our expectations due to the similar Romanian and Serbian cultural contexts. From Hofstede’s psycho-cultural model, Romania, and Serbia have a high-power distance and are collectivist, feminine, and short-term oriented (Burz and Marian, 2016; Milosevic, 2019), with similar life principles, which make them think and act relatively similarly. Despite the slight differences between power distance, individualism, and indulgence, we can sustain that our findings were cross-validated, and the path coefficients of the model were invariant across both samples.

### 4.1. Limitations and directions for future research

Beyond the strengths of this study, some limitations should be mentioned. First, the two samples are not big enough to allow generalizations and inferences about cross-cultural differences but can provide initial support in understanding how personality characteristics complement each other to enhance AE. Also, the structure of the samples was unbalanced, with women being much better represented than men. Previous findings showed small but significant gender differences in CSE, with a difference decreasing over time (e.g., Gang et al., 2020) and with greater contrast in Western than Eastern cultures (Gang et al., 2020). Regarding the impact on PsyCap, it seems that men’s PsyCap is higher than females (Jin et al., 2020). AE is also shaped by gender: women students are more engaged than men students in academic-related activities (Kessels and van Houtte, 2022) and exhibit greater AE than men (Babenko et al., 2018). Hence, further research needs to include balanced samples to identify the pattern of these interactions. Second, causal inferences could not be made because our research was cross-sectional. Third, the self-reported instruments may have affected students’ accuracy responses. Despite these limitations, the significance of the results should not be underestimated.

### 4.2. The theoretical and practical implications

Our study brings new information on COR theory about the role of two personality characteristics (i.e., CSE and PsyCap) and their relationships with an individual outcome (i.e., AE) in two different cultural and educational contexts (i.e., Romanian and Serbian). Also, this research is among the few which has worked with two personality characteristics together – CSE as trait-like and PsyCap as state-like (e.g., Tisu et al., 2020), to identify how they capitalize each other and increase students’ AE. From a practical perspective, our results can be helpful to both teachers and students, enhancing teaching-learning efficiency and academic well-being. For instance, positive self-assessment (i.e., high CSE) can function as a buffer, helping students to be assertive in dealing with and facing academic demands and challenges and utilizing them as a chance for future development (Leupold et al., 2020). Students high in CSE adapt more quickly to the stressful academic environment, are more engaged in learning tasks, and capitalize to a greater extent on personal resources (Haynie et al., 2017). Working on the two dimensions of CSE, enhancing self-esteem and self-efficacy, and lowering the level of neuroticism, CSE can be strengthened (Leupold et al., 2020). Former studies discovered that PsyCap contributes to AE both as an omnibus construct and through its four dimensions (e.g., Fang and Ding, 2020; Robayo-Tamayo et al., 2020). Consequently, to increase PsyCap, interventions can target the overall construct or each of its dimensions (e.g., Lupșa et al., 2020). For example, the PsyCap intervention (PCI) program includes exercises and coaching sessions that address the four components and facilitate PsyCap development. For each dimension taken separately, previous meta-analyses showed that interventions based on stress management are effective in increasing self-efficacy; those founded on the principles of positive psychology facilitate the growth of optimism and hope; and for increasing resilience, training focused on cognitive-behavioral approaches are efficacious (e.g., CareerSKILLS intervention) (Akkermans et al., 2015; Lupșa et al., 2020). Students high in PsyCap are more academically engaged than students with low PsyCap because of their self-confidence, optimism, hope in finding ways to work, and resilience in difficult situations (Siu et al., 2014). High self-efficacious students use the available resources to face the challenges in the academic environment, trust and persevere when encountering obstacles, and their optimism influences how they interpret events and, subsequently, adapt to the context (Azila-Gbettor et al., 2021).

## 5. Conclusion

Consistent with the COR theory (Hobfoll and Ford, 2007), personality and psychological resources are essential for students’ engagement, and the self’s involvement (i.e., CSE – self, and PsyCap – self in regards to the environment; Howard, 2017) can be considered a prerequisite for engagement experience. Therefore, the present research pointed out the importance of the CSE and PsyCap as two personality characteristics that support each other and increase students’ AE while also explaining the mediating mechanism of PsyCap. In addition, the results highlight those variables that can be intervened from an individual and organizational perspective to build a challenging and supportive learning environment that would increase the quality of the higher education teaching-learning process.

## Data availability statement

The raw data supporting the conclusions of this article will be made available by the correspondent author Delia Vîrgă delia.virga@e-uvt.ro, without undue reservation.

## Ethics statement

Ethical review and approval was not required for the study on human participants in accordance with the local legislation and institutional requirements. The patients/participants provided their written informed consent to participate in this study. All the procedures followed the ethical standards of the Scientific Council of University Research and Creation from West University of Timisoara (26093/05.05.2022) under the 1964 Helsinki Declaration and its later amendments or comparable ethical standards.

## Author contributions

RP has chosen the topic and contributed to the collection of the data, writing, and supervision of the present manuscript. EMS has contributed to the collection of the data, writing, and supervision of the present manuscript. DV has contributed to the design, methodology, writing, and supervision of the present manuscript. DP has contributed to the collection of the data and supervision of the present manuscript. All authors contributed to the article and approved the submitted version.

## Conflict of interest

The authors declare that the research was conducted in the absence of any commercial or financial relationships that could be construed as a potential conflict of interest.

## Publisher’s note

All claims expressed in this article are solely those of the authors and do not necessarily represent those of their affiliated organizations, or those of the publisher, the editors and the reviewers. Any product that may be evaluated in this article, or claim that may be made by its manufacturer, is not guaranteed or endorsed by the publisher.

## References

[ref1] Akkermans J. Nykänen M. Vuori J. (2015). “Practice makes perfect? Antecedents and consequences of an adaptive school-to-work transition” in Sustainable working lives - managing work transitions and health throughout the life course. eds. Vuori J. Blonk R. W. B. Price R. (London: Springer Publishers), 65–86.

[ref2] Alessandri G. Consiglio C. Luthans F. Borgogni L. (2018). Testing a dynamic model of the impact of psychological capital on work engagement and job performance. Career Dev. Int. 23, 33–47. doi: 10.1108/CDI-11-2016-0210

[ref3] Azila-Gbettor E. M. Mensah C. Abiemo M. K. Bokor M. (2021). Predicting student engagement from self-efficacy and autonomous motivation: a cross-sectional study. Cogent Educ. 8, 1942638, 1–1942614. doi: 10.1080/2331186X.2021.1942638

[ref4] Babenko O. Mosewich A. Abraham J. Lai H. (2018). Contributions of psychological needs, exhaustion in Canadian medical students. J. Educ. Eval. Health Prof. 15:2. doi: 10.3352/jeehp.2018.15.2 29307134 PMC5847840

[ref5] Bipp T. Kleingeld A. Ebert T. (2019). Core self-evaluations as a personal resource at work for motivation and health. Pers. Indiv. Differ. 151:109556. doi: 10.1016/j.paid.2019.109556

[ref6] Bowden J. L. H. Tickle L. Naumann K. (2021). The four pillars of tertiary student engagement and success: a holistic measurement approach. Stud. High. Edu. 46, 1207–1224. doi: 10.1080/03075079.2019.1672647

[ref7] Burz G. Marian L. (2016). Consideraţii privind coordonatele modelului psiho-cultural Hofstede în România [Considerations on coordinates of Hofstede type psycho-cultural model in Romania]. Rev. Manage. Econ. Eng. 15, 132–147.

[ref8] Byrne B. M. (2009). Structural equation modelling with AMOS: Basic concepts, programming, and applications (2). Mahwah, NJ: Erlbaum.

[ref9] Carmona-Halty M. Salanova M. Llorens S. Schaufeli W. B. (2021). Linking positive emotions and academic performance: the mediated role of academic psychological capital and academic engagement. Curr. Psychol. 40, 2938–2947. doi: 10.1007/s12144-019-00227-8

[ref10] Chang C. H. Ferris D. L. Johnson R. E. Rosen C. C. Tan J. A. (2012). Core self-evaluations: a review and evaluation of the literature. J. Manage. 38, 81–128. doi: 10.1177/0149206311419661

[ref11] Cheung G. W. Rensvold R. B. (2002). Evaluating goodness-of-fit indexes for testing measurement invariance. Struct. Eq. Model. 9, 233–255. doi: 10.1207/S15328007SEM0902_5

[ref12] Choi B. K. Rhee B. S. (2014). The influences of student engagement, institutional mission, and cooperative learning climate on the generic competency development of Korean undergraduate students. High. Educ. 67, 1–18. doi: 10.1007/s10734-013-9637-5

[ref13] Datu J. A. D. King R. B. Valdez J. P. M. (2018). Psychological capital bolsters motivation, engagement, and achievement: cross-sectional and longitudinal studies. J. Posit. Psychol. 13, 230–270. doi: 10.1080/17439760.2016.1257056

[ref14] Eysenck H. J. (1990). “Biological dimensions of personality” in Handbook of personality: Theory and research. ed. Pervin L. A. (New York: Guilford), 244–276.

[ref15] Fang S. Ding D. (2020). The efficacy of group-based acceptance and commitment therapy on psychological capital and school engagement: a pilot study among Chinese adolescents. J. Contextual Behav. Sci. 16, 134–143. doi: 10.1016/j.jcbs.2020.04.005

[ref16] Firouznia M. Hosseini S. H. Karamabad M. M. M. (2021). Affective-cognitive nature of engagement: correlating psychological capital and core-self-evaluations to work engagement via positive affects. Int. J. Proc. Manag. 14, 213–229. doi: 10.1504/IJPM.2021.113492

[ref17] Gang H. Anderson M. H. Summers J. (2020). Sex differences in Core self-evaluation: a Meta-analytic review. Acad. Manag. Proc. 1:17871. doi: 10.5465/AMBPP.2020.17871abstract

[ref003] Gavreliuc A. Gavreliuc D. (2018). Social cognitions and cultural dimensions in the Romanian educational field. J. Res. High. Educ. 2, 2–18. doi: 10.24193/JRHE.2018.2

[ref18] Gibson A. Hicks R. E. (2018). Psychological capital and core self-evaluations in the workplace: impacts on well-being. Int. J. Psychol. Stud. 10, 15–24. doi: 10.5539/ijps.v10n2p15

[ref19] Haynie J. J. Flynn C. B. Mauldin S. (2017). Proactive personality, core self-evaluations, and engagement: the role of negative emotions. Manag. Decis. 55, 450–463. doi: 10.1108/MD-07-2016-0464

[ref002] Hobfoll S.E. (2011). Conservation of resource caravans. doi: 10.1111/j.2044-8325.2010.02016.x

[ref20] Hobfoll S. E. Ford J. S. (2007). “Conservation of resources theory” in Encyclopedia of stress. ed. Fink G.. 2nd ed. (Elsevier: Academic Press), 562–567. doi: 10.1016/B978-012373947-6.00093-3

[ref001] Hobfoll S. E. Halbesleben J. Neveu J. P. Westman M. (2018). Conservation of resources in the organizational context: the reality of resources and their consequences. Annu. Rev. Organ. 5, 103–128. doi: 10.1146/annurev-orgpsych-032117-104640

[ref21] Hofstede G. Hofstede G. J. Minkov M. (2010). Cultures and organizations: Software of the mind. (3rd ed.). New York: McGraw-Hill.

[ref22] Howard M. C. (2017). The empirical distinction of core self-evaluations and psychological capital and the identification of negative core self-evaluations and negative psychological capital. Pers. Indiv. Differ. 114, 108–118. doi: 10.1016/j.paid.2017.03.061

[ref23] Ivanović M. Ivanović U. (2018). The most important self-evaluation and self-efficacy in choosing a vocational as determinants of vocational outcomes of junior karatekas. Homo Sporticus 20, 5–10.

[ref24] Jin J. Li H. Song W. Jiang N. Zhao W. Wen D. (2020). The mediating role of psychological capital on the relation between distress and empathy of medical residents: a cross-sectional survey. Med. Educ. Online 25:1710326. doi: 10.1080/10872981.2019.1710326 31900104 PMC6968582

[ref25] Judge T. A. Erez A. Bono J. E. Thoresen C. J. (2003). The core self-evaluations scale: development of a measure. Pers. Psychol. 56, 303–331. doi: 10.1111/j.1744-6570.2003.tb00152.x

[ref26] Judge T. A. Hurst C. (2007). Capitalizing on one's advantages: role of core self-evaluations. J. Appl. Psychol. 92, 1212–1227. doi: 10.1037/0021-9010.92.5.1212 17845081

[ref27] Kahu E. R. Picton C. Nelson K. (2020). Pathways to engagement: a longitudinal study of the first-year student experience in the educational interface. High. Educ. 79, 657–673. doi: 10.1007/s10734-019-00429-w

[ref28] Kessels U. van Houtte M. (2022). Side effects of academic engagement? How boys’ and girls’ well-being is related to their engagement and motivational regulation. Gender Educ. 34, 627–642. doi: 10.1080/09540253.2021.2011840

[ref29] Ketonen E. E. Malmberg L. E. Salmela-Aro K. Muukkonen H. Tuominen H. Lonka K. (2019). The role of study engagement in university students' daily experiences: a multilevel test of moderation. Learn. Individ. Differ. 69, 196–205. doi: 10.1016/j.lindif.2018.11.001

[ref30] Lei H. Cui Y. Zhou W. (2018). Relationships between student engagement and academic achievement: a meta-analysis. Soc. Behav. Personal. 46, 517–528. doi: 10.2224/sbp.7054

[ref31] Leupold C. R. Lopina E. C. Erickson J. (2020). Examining the effects of core self-evaluations and perceived organizational support on academic burnout among undergraduate students. Psychol. Rep. 123, 1260–1281. doi: 10.1177/0033294119852767 31132928

[ref32] Little T. D. Cunningham W. A. Shahar S. Widaman K. F. (2002). To parcel or not to parcel: exploring the question, Weighing the Merits. Struct. Eq. Model. 9, 151–173. doi: 10.1207/S15328007SEM0902_1

[ref33] Lupșa D. Vîrgă D. (2018). Psychological capital questionnaire (PCQ): analysis of the Romanian adaptation and validation. Psihologia Resurselor Umane [Psychol. Hum. Resour.] 16, 27–39. doi: 10.24837/pru.2018.1.484

[ref34] Lupșa D. Vîrga D. Maricuțoiu L. P. Rusu A. (2020). Increasing psychological capital: a pre-registered Meta-analysis of controlled interventions. Appl. Psychol. 69, 1506–1556. doi: 10.1111/apps.12219

[ref35] Luthans F. Avolio B. J. Avey J. B. Norman S. M. (2007). Positive psychological capital: measurement and relationship with performance and satisfaction. Pers. Psychol. 60, 541–572. doi: 10.1111/j.1744-6570.2007.00083.x

[ref36] Luthans K. W. Luthans B. C. Palmer N. F. (2016). A positive approach to management education: the relationship between academic PsyCap and student engagement. J. Manag. Dev. 35, 1098–1118. doi: 10.1108/JMD-06-2015-0091

[ref37] Luthans F. Youssef-Morgan C. M. (2017). Psychological capital: an evidence-based positive approach. Ann. Rev. Organ. Psych. 4, 339–366. doi: 10.1146/annurev-orgpsych-032516-113324

[ref38] Ma Y. Qian Z. Zhong L. (2022). Influence of Core self-evaluations on work engagement: the mediating role of informal field-based learning and the moderating role of work design. Sustainability 14:5319. doi: 10.3390/su14095319

[ref39] Martínez I. M. Youssef-Morgan C. M. Chambel M. J. Marques-Pinto A. (2019). Antecedents of academic performance of university students: academic engagement and psychological capital resources. Educ. Psychol. 39, 1047–1067. doi: 10.1080/01443410.2019.1623382

[ref40] Milosevic D. (2019). A comparison of Hofstede cultural dimensions: Italy, Germany and Serbia. Econ. Manag. Nat. Resour., 1–8.

[ref41] Nolzen N. (2018). The concept of psychological capital: a comprehensive review. Manag. Rev. Q. 68, 237–277. doi: 10.1007/s11301-018-0138-6

[ref42] Ouweneel E. LeBlanc P. M. Schaufeli W. B. (2011). Flourishing students: a longitudinal study on positive emotions, personal resources, and study engagement. J. Posit. Psychol. 6, 142–153. doi: 10.1080/17439760.2011.558847

[ref43] Paloş R. Maricuţoiu L. P. Costea I. (2019). Relations between academic performance, student engagement, and student burnout: a cross-lagged analysis of a two-wave study. Stud. Educ. Eval. 60, 199–204. doi: 10.1016/j.stueduc.2019.01.005

[ref44] Petrović I. B. Vukelić M. Čizmić S. (2017). Work engagement in Serbia: psychometric properties of the Serbian version of the Utrecht work engagement scale (UWES). Front. Psychol. 8:1799. doi: 10.3389/fpsyg.2017.01799 29085319 PMC5650702

[ref45] Robayo-Tamayo M. Blanco-Donoso L. M. Román F. J. Carmona-Cobo I. C. Moreno-Jiménez B. Garrosa E. (2020). Academic engagement: a diary study on the mediating role of academic support. Learn. Individ. Differ. 80:101887. doi: 10.1016/j.lindif.2020.101887

[ref46] Rogers W. M. Schmitt N. (2004). Parameter recovery and model fit using multidimensional composites: a comparison of four empirical parceling algorithms. Multivar. Behav. Res. 39, 379–412. doi: 10.1207/S15327906MBR3903_1

[ref47] Rosenberg M. (1965). Society and the adolescent self-image. Princeton, NJ: Princeton University Press.

[ref004] RStudio Team. (2020). RStudio: Integrated Development for R, RStudio, PBC, Boston, MA. Available at: http://www.rstudio.com/.

[ref48] Sava S. L. Vîrgă D. Paloş R. (2020). The role of teacher support, students’ need satisfaction, and their psychological capital in enhancing students’ self-regulated learning. Stud. Psychol. 62, 44–57. doi: 10.31577/sp.2020.01.790

[ref49] Schaufeli W. B. Bakker A. B. Salanova M. (2006). The measurement of work engagement with a short questionnaire: across-national study. Educ. Psychol. Meas. 66, 701–716. doi: 10.1177/0013164405282471

[ref50] Schaufeli W. B. Martínez I. M. Marques Pinto A. Salanova M. Bakker A. B. (2002). Burnout and engagement in university students: a cross-national study. J. Cross-Cult. Psychol. 33, 464–481. doi: 10.1177/0022022102033005003

[ref51] Schwarzer R. Jerusalem M. (1995). “Generalized self-efficacy scale” in Measures in health psychology: A user’s portfolio. Causal and control beliefs. eds. Weinman J. Wright S. Johnston M. (Windsor: NFER-NELSON), 35–37.

[ref52] Shernoff D. J. Csikszentmihalyi M. Schneider B. Shernoff E. S. (2003). Student engagement in high school classrooms from the perspective of flow theory. School Psychol. Quart. 18, 158–176. doi: 10.1007/978-94-017-9094-9_24

[ref53] Shih S. (2008). The relation of self-determination and achievement goals to Taiwanese eighth graders’ behavioral and emotional engagement in schoolwork. Elem. School J. 108, 313–334. doi: 10.1086/528974

[ref54] Siu O. L. Bakker A. B. Jiang X. (2014). Psychological capital among university students: relationships with study engagement and intrinsic motivation. J. Happiness Stud. 15, 979–994. doi: 10.1027/1866-5888/a000092

[ref55] Stoeber J. Childs J. H. Hayward J. A. Feast A. R. (2011). Passion and motivation for studying: predicting academic engagement and burnout in university students. Educ. Psychol. 31, 513–528. doi: 10.1080/01443410.2011.570251

[ref56] Tims M. Akkermans J. (2017). Core self-evaluations and work engagement: testing a perception, action, and development path. PLoS ONE 12:e0182745. doi: 10.1371/journal.pone.0182745 28787464 PMC5546708

[ref57] Tisu L. Lupșa D. Vîrgă D. Rusu A. (2020). Personality characteristics, job performance and mental health: the mediating role of work engagement. Pers. Indiv. Differ. 153:109644. doi: 10.1016/j.paid.2019.109644

[ref58] Vîrgă D. Pattusamy M. Kumar D. P. (2020). How psychological capital is related to academic performance, burnout, and boredom? The mediating role of study engagement. Curr. Psychol. 41, 6731–6743. doi: 10.1007/s12144-022-03339-w

[ref59] Yan X. Yang K. Su J. Luo Z. Wen Z. (2018). Mediating role of emotional intelligence on the associations between Core self-evaluations and job satisfaction, work engagement as indices of work-related well-being. Curr. Psychol. 37, 552–558. doi: 10.1007/s12144-016-9531-2

[ref60] You J. W. (2016). The relationship among college students' psychological capital, learning empowerment, and engagement. Learn. Individ. Differ. 49, 17–24. doi: 10.1016/j.lindif.2016.05.001

